# Normalization with Corresponding Naïve Tissue Minimizes Bias Caused by Commercial Reverse Transcription Kits on Quantitative Real-Time PCR Results

**DOI:** 10.1371/journal.pone.0167209

**Published:** 2016-11-29

**Authors:** Andreas Garcia-Bardon, Serge C. Thal

**Affiliations:** Department of Anesthesiology, Medical Center of the Johannes Gutenberg University, Mainz, Germany; Wayne State University, UNITED STATES

## Abstract

Real-time reverse transcription polymerase chain reaction (PCR) is the gold standard for expression analysis. Designed to improve reproducibility and sensitivity, commercial kits are commonly used for the critical step of cDNA synthesis. The present study was designed to determine the impact of these kits. mRNA from mouse brains were pooled to create serial dilutions ranging from 0.0625 μg to 2 μg, which were transcribed into cDNA using four different commercial reverse-transcription kits. Next, we transcribed mRNA from brain tissue after acute brain injury and naïve mice into cDNA for qPCR. Depending on tested genes, some kits failed to show linear results in dilution series and revealed strong variations in cDNA yield. Absolute expression data in naïve and trauma settings varied substantially between these kits. Normalization with a housekeeping gene failed to reduce kit-dependent variations, whereas normalization eliminated differences when naïve samples from the same region were used. The study shows strong evidence that choice of commercial cDNA synthesis kit has a major impact on PCR results and, consequently, on comparability between studies. Additionally, it provides a solution to overcome this limitation by normalization with data from naïve samples. This simple step helps to compare mRNA expression data between different studies and groups.

## Introduction

Real-time reverse transcription polymerase chain reaction (PCR) is the method of choice for fast and highly accurate quantitative assessment of gene expression [1]. It is more sensitive than the Northern Blot analysis or the conventional reverse transcription PCR [2]. Further, it allows high throughput at relatively low cost. Although Higuchi et al. developed this technique as early as 1993 [3], the question to correct data normalization is still subject to frequent criticism and remains problematic. Guénin et al. demonstrated the need to perform a systematic condition-specific validation of factors that influence PCR results [4]. A variety of criteria were developed to minimize the impact of the experimental setup on results [5, 6]. These include: (a) quality and quantity control of RNA [5, 7]; (b) optimal reverse transcription reaction; and (c) proper selection of endogenous control genes for normalization of run-to-run variations [8, 9]. The impact of reverse transcription, the generation of complimentary DNA (cDNA) from messenger RNA (mRNA) transcripts, on PCR results has not been addressed in detail yet.

The first aim of this study was to determine if quantification data would be significantly different depending on the cDNA synthesis kits. The second aim was to find out whether additional steps would help to overcome such limitations. To investigate this question, we selected four commercial cDNA synthesis kits for reverse transcription of identical samples under different experimental conditions to evaluate the impact on selected example PCR targets before and after normalization with housekeeping genes and, in an additional normalization step, using corresponding naïve tissue samples.

## Material and Methods

### Animals

A total of 16 male C57Bl6N mice (weight 20 g to 24 g, Charles River Laboratory, Sulzfeld, Germany) were investigated. Before and during experiments, all animals were cared for in compliance with institutional guidelines of the Johannes Gutenberg University, Mainz, Germany. All experiments were performed in accordance with the German Animal Welfare Act (TierSchG) and approved by the Animal Ethics Committee of the “Landesuntersuchungsamt Rheinland-Pfalz” (LUA), the State Agency for Consumer and Health Protection (protocol number: G07-1-021).

### Traumatic brain injury

The animals were anesthetized with isoflurane in an air mixture (40% O_2_ and 60% N_2_) via facemask in spontaneously breathing animals. Anesthesia was terminated at the end of surgery by discontinuing isoflurane application. The brain trauma model was performed as previously described [10]. Animals were placed in a stereotactic frame (Kopf Instruments, Tujunga, CA, USA) and were subjected to controlled cortical impact (CCI). A craniotomy (4x4 mm) was performed, using a high-speed drill over the right parietal cortex between the sagittal, lambdoid, and coronal sutures. The tip of a custom pneumatically-controlled cortical impactor (L. Kopacz, Mainz, Germany) was placed directly onto the brain surface. For all animals, we used an impactor tip of 3 mm in diameter, an impact velocity of 8 m/s, an impact duration of 150 msec and a brain penetration of 1 mm. Immediately after the cortical impact, the craniotomy was closed with the initially removed bone flap and fixed with conventional tissue glue (Histoacryl, Braun-Melsungen, Melsungen, Germany). The wounds were closed with filament sutures at the end of surgery.

Rectal temperature was maintained at a constant 37°C by using a feedback-controlled heating pad (Hugo Sachs, March-Hugstetten, Germany). After preparation, all animals were placed in individual cages and allowed to recover for 2 hours in an incubator maintained at 33°C and a humidity of 35% (IC8000, Dräger, Lübeck, Germany).

### Experimental protocols

1. Analysis of linearity of cDNA synthesis by use of pooled mRNA from naïve brain tissue:
  Target Genes: iNOS, cyclophilin A, β_2_-microglobulin
2. B. Analysis of impact of kit selection on expression data by use of mRNA from brain tissue of healthy and brain-injured male mice (n = 5/group):
  Target genes: iNOS, IL-1β, cyclophilin A

#### Details for protocol A

After the quality control and the pooling of the extracted RNA, we created a 6-step dilution series (at concentrations of: 2 μg/μL, 1 μg/μL, 0.5 μg/μL, 0.25 μg/μL, 0.125 μg/μL, 0.0625 μg/μL). Subsequently, 1 μL of each sample was reverse-transcribed into cDNA by four kits (see **Table 1** for detailed description of kits). We used each kit according to the manufacturer’s instructions. Kit 1 and kit 3 allowed a reverse transcription with a mix of oligo-dT primers and random hexamer primers. Kit 4 contained a predefined mix of both primers, while the manufacturer’s protocol of kit 2 suggested to only use random hexamer primers. To ensure better comparability between the kits used, and to prevent enzymatic interactions, none of the samples were treated with DNAse. **Table 1** shows the range of linear reaction of each kit, as stated by the respective manufacturers. We found that the highest RNA amount, 2 μg, exceeded the upper limit specified for kit 1 and kit 2, while the lowest RNA amount, 0.0625 μg, remained below the lower limit of kit 3. For all dilutions, we used DNA LoBind Tubes (Eppendorf AG, Hamburg, Germany) to minimize RNA adhesion to the tube walls.

**Table 1:** Description of cDNA synthesis kits.

Kit name, manufacturer	Catalogue no.	Reverse transcriptase	RNase H activity	Primer	RNA range
Kit 1: Verso^TM^ cDNA Kit, Thermo Scientific (Waltham, MA, USA)	AB1453A	not mentioned	reduced	oligo-dT + random hexamers	1 pg—1 μg
Kit 2: Superscript^®^ ViloTM cDNA Synthesis Kit, Invitrogen (Carlsbad, CA, USA)	not provided	engineered version of m-MLV RT	reduced	Random	< 2.5 μg
Kit 3: Transcriptor High Fidelity cDNA Synthesis Kit, Roche (Mannheim, DEU)	05081955001	not mentioned	not mentioned	oligo-dT + random hexamers	1 ng—4 μg
Kit 4: Quantitect^®^ Reverse Transcription Kit, Qiagen (Hilden, DEU)	205311	not mentioned	not mentioned	Premixed primers	10 pg—1 μg

All applied cDNA synthesis kits, primers and RNA range were used according to the manufacturers’ instructions.

#### Details for protocol B

To investigate the impact of cDNA synthesis on results, we did not pool the extracted RNA prior to conducting the analysis. We separately reverse-transcribed 1 μg of each sample using each of the four kits under the same conditions, as described in protocol A. The study was designed to quantify post-injury mRNA expression of two regulated genes (inflammatory marker genes) and one non-regulated gene (housekeeping gene).

### Tissue Preparation

Both healthy animals and animals subjected to experimental traumatic brain injury were sacrificed by cervical dislocation under isoflurane anesthesia. Then, the brain tissue was removed carefully and placed in a cooled (6°C) brain matrix. A 3-mm thick slice was cut in coronal orientation and separated in quadrants. The right upper regions were collected, frozen in liquid nitrogen and stored at -80°C.

### RNA isolation, quality control and cDNA synthesis

For RNA extraction, brain tissue samples were lysed with RLT-buffer (Qiagen, Hilden, Germany) and homogenized with a MM300 mill mixer (Retsch, Haan, Germany) at 30 Hz for 2 minutes. Then, we isolated the total RNA from the lysed and homogenized cells by using a RNeasy Lipid Tissue Mini Kit (Qiagen) according to the manufacturer's instructions and eluted these with 30 μL to 50 μL RNase-free water. RNA quality was confirmed via gel electrophoresis (Experion Automated Electrophoresis System, Bio-Rad, Munich, Germany) for RQI (RNA quality indicator) ≥8 [11]. We calculated the RNA concentration, using a Quanti-iT™ RNA Assay kit with the Qubit™ fluorimeter (Molecular Probes™, Thermo-Fisher Scientific, Germany) and by spectral absorption (A260 nm/A280 nm; NanoVue, General Electrics, MA, USA). Most scientist worldwide use the A260/A280 absorption method to check for e.g. protein contamination and purity of the RNA with a ratio of ~2.0 generally accepted as “pure RNA”. Abnormal 260/280 ratios usually indicate contamination by proteins or reagents such as phenol. DNA/RNA shreds and single nucleotides also contribute to the absorption at A260 and may serve as confounding factor. To overcome this limitation specific assays were developed with RNA-binding dyes. The Quant-iT™ RNA assay was designed to specifically quantify RNA, rRNA, or mRNA in a range from 250 pg/μL to 100 ng/μL (excitation/emission maxima 644/673 nm). Unlike UV absorbance measurements at 260 nm, the reagent does not detect significant sample contamination by free nucleotides. Thus, the Quant-iT™ RNA assay more accurately measures the amount of intact RNA polymers in potentially degraded samples. Unfortunately, detailed information on dye, specificity for RNA, and performance compared with other dyes, e.g. RiboGreen [12], are not available for the general public due to strict confidentiality policy of the manufacturer.

### Quantitative polymerase chain reaction (real-time PCR)

We amplified equal amounts of cDNA (1 μL) of each sample in triplicates, using the real-time Lightcycler 480 PCR System (Roche). **Table 2** shows the real-time cycling parameters and applied primers and probes. We used mRNA-specific primers for real-time PCR. Each run was designed to contain samples of all points in time and regions to minimize the influence of run-to-run variations. We performed a total of six runs for each gene product.

**Table 2:** Primer and probes with optimized temperature conditions for real-time PCR.

Polymerase chain reaction assay	Oligonucleotide sequence	GeneBank no.
[Amplicon size, annealing temperature, A, E]	(5´-3´)	
cyclophilin A (PPIA)	Forw: 5´-GCGTCTSCTTCGAGCTGTT-3´	NM_008907
*[146 bp*, *55°C*, *A*: *10 s*, *E*: *15 s]*	Rev: 5´-RAAGTCACCACCCTGGCA-3´	
	FL: 5´-GCTCTGAGCACTGGRGAGAAAGGA-FL	
	Cy5: Cy5-TTGGCTATAAGGGTTCCTCCTTTCACAG-Phos	
inducible nitric oxide synthase (iNOS)	Forw: 5’-TGTGTCAGCCCTCAGAGT AC-3’	NM_010927
[312 bp, 55°C, A: 20 s, E: 30 s]	Rev: 5’-CACTgACACTYCgCACAA-3’	
	FL: 5'-gAAgCCCCgCTACTACTCCATC-FL	
	Red640: Red640-GCTCCTCCCAGGACCACACCC-Phos	
beta-2-microglobulin (B2m)	Forw: 5'-CCGAACATACTGAACTGC-3’	NM_009735
[101 bp, 55°C, A: 20 s, E: 15 s]	Rev: 5'-AGAAAGACCAGTCCTTGC-3’	
	FL: 5'-GAAGCCCCGCTACTACTCCATC-FL-3’	
	Red670: Red670-AAATCCAAATGCTGAAGAACGGGA-Phos	

Forw, sense primer; Rev, antisense primer; Cy5, Cyanine 5; Phos, Phosphate; FL, fluorescein; A, annealing time; E, extension time.

### Statistical Analysis

We compared the mRNA expression data between all experimental groups by means of the Mann-Whitney U test and adjusted the p-values for multiple comparisons (Bonferroni adjustment). We also tested the correlation between absolute gene expression and the RNA concentration with the Spearman rank-order correlation. Results are presented as mean ± standard deviation (S.D.). Differences were considered significant at p<0.05. We performed a statistical analysis with the Prism 6 Statistical Software package (GraphPad Software, La Jolla, CA, USA).

## Results

### Influence of kit selection on linearity and efficiency of cDNA synthesis

**Fig 1:**
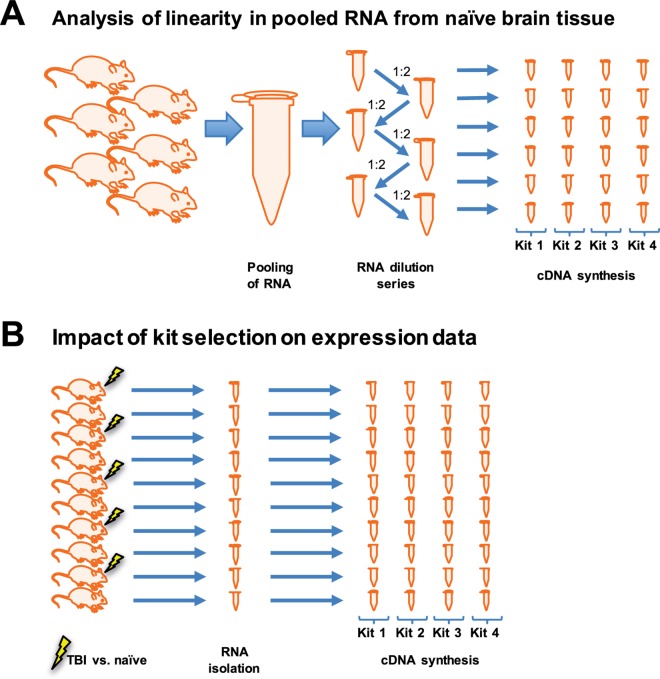
Schematic presentation of the study design. **A)** RNA of brain tissue from six naïve animals was extracted and pooled to create an identical dilution series. cDNA synthesis was then performed with each of the four kits. **B)** RNA of brain tissue from 6 naïve animals and 6 animals suffering traumatic brain injury was extracted. Each sample was reverse-transcribed, using all four kits, again ensuring the exact same starting material for each kit.

**Fig 2:**
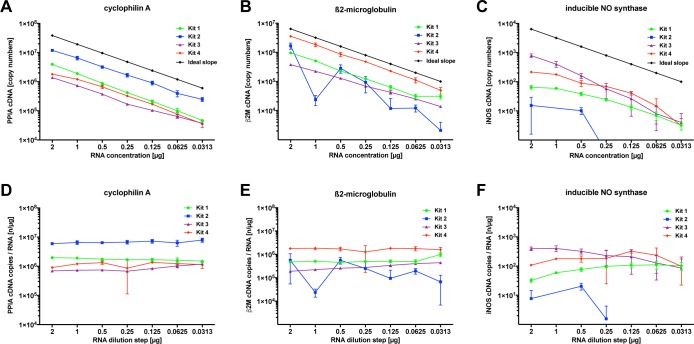
Influence of kit selection on linearity and efficacy of cDNA synthesis. The figure depicts absolute cDNA copy numbers after reverse transcription of one mRNA dilution series (pooled from mRNA of six naïve animals) with four different cDNA synthesis kits and RT-qPCR for the housekeeping genes ß2M and PPIA and the low-copy, regulated gene iNOS. All four kits show a linear correlation between the mRNA concentration and the cDNA output for ß2M and PPIA (Spearman's rank-order correlation coefficient; r^2^>0.99), except at a ß2M mRNA concentration of 1μg. Only kit 3 shows a strong linear correlation between iNOS cDNA and mRNA (r^2^>0.99; c). The mRNA/cDNA ratio varies significantly between the individual kits and the genes, although the individual kits show a certain consistency regarding the single gene dilution series (d-f). All plots show mean ± S.D.

To determine the efficacy of reverse transcription in terms of linearity and the amount of transcribed cDNA products, we processed serial diluted samples from a pooled mRNA stock with each kit (for a schematic presentation, see **Fig 1A**). Real-time PCR for the housekeeping gene Cyclophilin A showed a nearly linear concentration-dependent increase in the copy number, which was close to the ideal dilution curve in all kits tested (see **Fig 2A**). A good correlation, on the other hand, was present in all kits between mRNA and cDNA for this highly abundant gene, with kit 2 yielding a significantly higher overall cDNA content of up to 10^5^–fold, compared to kits 1, kit 3, and kit 4. PCR quantification for transcripts of β_2_-microglobulin revealed a completely different picture (see **Fig 2B**). For this gene, only kit 3 and kit 4 showed linear concentration-dependent changes in all dilution steps. Kit 1 showed strong variations in low concentrations and kit 2 completely failed to show linear results with reproducible PCR failure in repeated runs at 1 μg mRNA concentration. To test the efficacy of reverse transcription in the low copy range, we selected iNOS as low-abundant enzyme. After employing PCR, only kit 1 and kit 3 showed a nearly linear correlation between the copy number and the mRNA concentration, whereas kit 2 failed to produce cDNA at mRNA concentrations below 0.5 μg (see **Fig 2C**). To test whether the efficacy of reverse transcription is independent of the investigated target gene, we calculated the ratio between the target gene copy number and the mRNA concentration (see **Fig 2D–2F**). This analysis demonstrates that the efficacy of the cDNA synthesis is not similar within single kits, but varies substantially between the investigated target genes. In summary, depending on the target gene, the tested kits in most, but not all cases, showed a linear RNA cDNA correlation.

### Impact of kit selection on expression data in an experimental paradigm of brain injury

**Fig 3:**
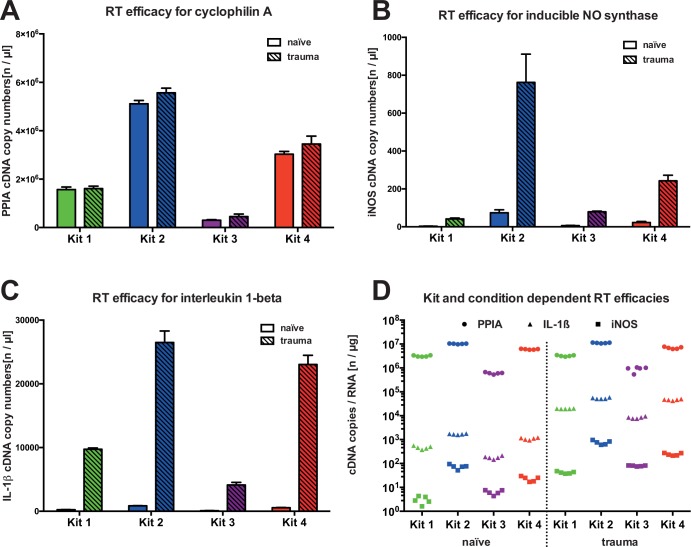
Influence of kit selection on absolute copy numbers. This figure demonstrates absolute cDNA copy numbers from naïve (n = 5) vs. trauma (n = 5) samples for PPIA (high-copy, non-regulated gene), iNOS and IL-1β (both low-copy, regulated genes). An equal amount of each sample was reverse-transcribed, using all four kits. A significantly higher cDNA expression was present in trauma vs. naïve samples for PPIA (kit 2, p = 0.002; kit 3, p = 0.014; and kit 4, p = 0.027), iNOS and IL-1β (all kits, p<0.05). Absolute and relative results vary significantly between the kits used (a-c). In accordance with our previous findings, the mRNA/cDNA ratio varies substantially between the kits (d) (although identical starting material was used) and between genes (although reverse-transcribed by the same kit under equal experimental conditions). All bar charts show mean ± S.D.

To test the impact of commercial kit selection on experimental PCR results, we used each of the four kits tested to reverse-transcribe the same set of mRNA samples from a standardized brain injury paradigm and from corresponding naïve brain tissue samples (for a schematic presentation, see **Fig 1B**). To avoid influence of freeze-thaw cycles, we prepared mRNA aliquots before the testing to rule out run-to-run variations. At first, we analyzed the stable expressed control gene, cyclophilin A. We found that cDNA produced by the four kits tested, showed significant different absolute cyclophilin A copy numbers, e.g., kit 2 produced almost 17 times more cDNA compared to kit 3 (see **Fig 3A**). Although generally accepted as a valid housekeeping gene after TBI, cyclophilin A copy numbers varied within some kits between naïve vs. trauma samples, e.g., kit 2 and kit 3. As expected, for iNOS and IL-1β, the amount of cDNA increased significantly in trauma samples compared to naïve samples, regardless of the selected kit (see **Fig 3B and 3C**). With both genes, kit 2 and kit 4 produced significantly larger amounts of cDNA in naïve and in trauma samples compared to the other kits. To test whether the efficacy of a reverse transcription is independent of the investigated target gene and investigated tissue, we calculated the ratio between the copy number of the target gene and the mRNA concentration (see **Fig 3D**). In line with the previously described findings, the efficacy of the cDNA synthesis varies substantially between the investigated target genes and the single kits, although we provided each kit with identical starting material.

### Influence of normalization step with a housekeeping gene on expression data

**Fig 4:**
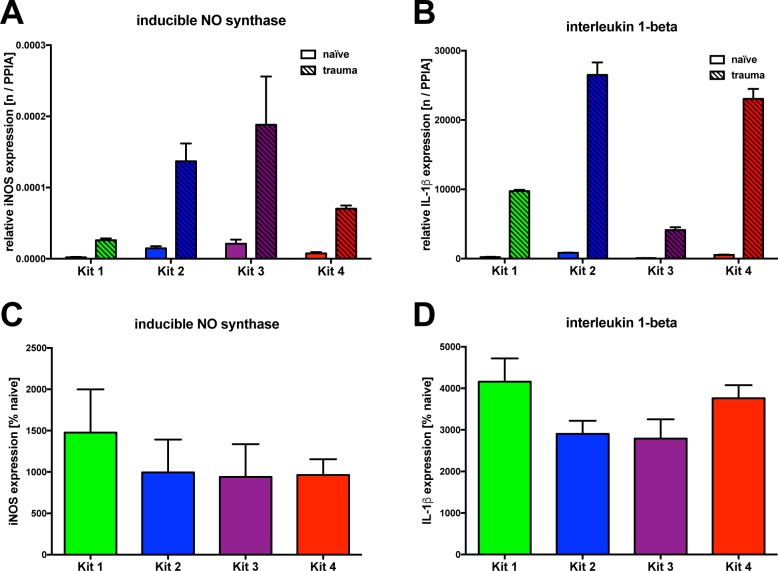
Impact of kit selection on expression data in an experimental paradigm of brain injury. To demonstrate whether discrepancies in the efficacy of cDNA synthesis between the four kits influence the results after normalization, the regulated genes iNOS and IL-1β were normalized against the non-regulated PPIA. Although equal amounts of mRNA were used, even after normalization, results for iNOS and IL-1β vary significantly (a, b). After calculating the percentage of normalized trauma cDNA from normalized naïve cDNA for each kit, no significant difference between the kits could be shown neither for iNOS nor for IL-1β (c, d) (p = n.s.). All bar charts show mean ± S.D.

To this day, the normalization of real-time PCR data has been performed using one or more internal control genes or housekeeping genes to eliminate run-to-run variations. We used this strategy, both to investigate if this step results in similar relative expression data and to see if this approach sufficiently eliminates the difference in absolute copy numbers or relative data. We found that the expression of iNOS and IL-1β were normalized with cyclophilin A (see **Fig 4A and 4B**). Although we used the same cDNA samples from five naïve or five post-TBI mice with each of the four kits, relative expression data were completely different. We found statistically significant differences for both iNOS and IL-1β, regardless of whether naïve or trauma material was used. This means, on the one hand, that the relative expression data is highly dependent on the selected reverse-transcription kit and, on the other hand, that the difference between the kits is not sufficiently corrected by normalization with a housekeeping gene, explaining to some extent the difference in expression between different studies.

### Influence of normalization step with a naïve sample data on kit-dependent expression

We observed that the extent of normalized expression data differed markedly between the kits. However, comparing the magnitude of expression in naïve and trauma data appeared to be similar within a single kit. In order to improve both the similarity and the comparability between results obtained by different kits, we set out to test a new approach. We added an additional normalization step, using housekeeping gene corrected data obtained both from trauma and naïve animals. Each data point of trauma expression was divided by the mean of all five normalized expression data obtained from naïve animals. The calculated data show the data as percentage of naïve tissue. For both iNOS (see **Fig 4C**) and IL-1β (see **Fig 4D**) we found that this method is able to reduce kit-dependent differences compared to a normalization with only the housekeeping gene being used. Our data suggest that relative data normalized with naïve tissue helps to overcome limitations by the use of different kits and, further, improves the comparability of expression data between different studies.

## Discussion

This study has yielded the following results: (1.) irrespective of the kit selected, mRNA and transcribed cDNA showed a linear correlation between the genes tested; (2.) depending on the choice of the cDNA synthesis kit, the absolute gene expression is subject to considerable variations, e.g., cyclophilin A varied 10^5^-fold, thus, clearly demonstrating the limitation to compare absolute gene expression data determined by different kits; (3.) after normalization with a non-regulated housekeeping gene, cDNA yields still varied substantially; (4.) normalization by use of normalized expression data obtained from naïve samples of identical brain regions and sample sizes markedly reduced variations between kits.

An important prerequisite of quantitative PCR is the reliability to detect small expression changes with high specificity for selected transcripts. In order to improve reproducibility, most laboratories replaced self-build components with commercially available kits. Unfortunately, there is only limited independent evidence available regarding the efficacy and comparability of commercial kits. A recent publication found substantial differences in the performance of PCR assays [13], indicating that the kit selection could have a major impact on study results. To allow a comparison of quantitative data, either between different research teams, publications or even within laboratories, we believe that the essential steps of PCR need to become more reproducible.

Experimental variation of PCR to quantify mRNA can mainly be attributed to reverse transcription. In this step, a reverse transcriptase (RT) is used to produce single-stranded complementary DNA (cDNA) from template mRNA. The synthesized cDNA can then be quantified by PCR. The use of an efficient, high quality RT is critical to obtain cDNA that contains the full transcript as a basis for subsequent analyses.

A second important component for a successful reverse transcription is the RNA priming strategy. For this purpose, laboratories most commonly use oligo(dt), random primers or a combination of both, each having different benefits and drawbacks. Oligo(dt) primers, for instance, show a high specificity for mRNA. However, their difficult secondary structure may lead to an incomplete cDNA synthesis, as they always initiate reverse transcription at the polyA end of the transcript. Priming of 18S rRNA or fragmented RNA may be problematic. Random primers anneal throughout the mRNA strand and, therefore, increase the cDNA yield, but also tend to overestimate copy numbers when used in real-time PCR. Our findings show that primer selection has to be adjusted to the specific reverse transcriptase. Furthermore, to ensure efficient cDNA synthesis, optimal experimental conditions, e.g., the right temperature have to be created.

Commercially available cDNA synthesis kits are designed to provide optimal interaction between each component, including primers and reverse transcriptase. Manufacturers offer different options: Table 1 shows that kit 1 and kit 3 are designed to use oligo(dT) or random primers, while kit 2 includes only random primers and kit 4 is equipped with an unspecified combination of premixed primers. To ensure comparability between these kits, random primers where used in kit 1 through kit 3.

The reason for choosing a specific reverse transcriptase kit is most often not further described in research publications. As there is a great number of commercially available kits, we only chose those four kits which we use most frequently in our laboratory. However, we assume that our results can be translated into other kits due to the existing similarities between the RT-kits available on the market. Even in this small group, the impact on quantification was substantial and suggests that the RT procedure is a greatly underestimated and confounding factor.

This is a surprising result, since this normalization strategy is thought to be sufficient to eliminate all run-to-run variations, if: (a) both a comparable amount of starting material and an equal amount of mRNA are used for each samples [14]; and (b) if an internal standard, known to be expressed at a constant level and unaffected by the experimental treatment, is used as control gene [5, 15, 16]. We found that only if normalized results were divided by each other (mean of experimental condition/mean of naïve), all kits delivered similar results. Consequently, these results challenge the comparability of gene quantification studies and raise the question whether the current practice of presenting the data as “relative gene expression” only after performing a normalization with a housekeeping gene does really reflect the “true” gene expression or if the results are biased by the choice of the mRNA transcription kit.

We believe that, in order to minimize the influence of the reverse transcription on study outcomes, results should be presented as percentage of naïve. Only the relative increase in gene expression compared with naïve has shown to be a solid basis for discussing and comparing results, and is powerful enough to overcome study-to-study variations.

These findings also demonstrate the need to confirm the suitability of RT-kits for the target genes selected. Although the ß2M dilution series was repeatedly reverse-transcribed with kit 2, no cDNA copies could be detected by PCR at 1 μg RNA. There seems to be a critical mass of inhibiting factors at this concentration, impeding successful cDNA synthesis or detection. To minimize a potential influence on results and to obtain valid results, we recommend to critically evaluate the combination of the genes of interest and the chosen RT kit. Furthermore, the results demonstrate a highly variable efficacy of the RT step, reflected in the ratio of both the mRNA concentration and the cDNA copy number. A gene-dependent reverse transcription efficacy clearly shows the limitation of relative quantification, if relative crossing point (Ct) values of genes with different efficacies are used for normalization.

The use of anesthesia as a form of euthanasia is also a confounding factor that influences gene expression analysis [17]. Even a few minutes of general anesthesia seem to significantly alter cerebral mRNA expression levels. Irrespective of any normalization strategy applied, when comparing qPCR data, we believe it to be essential to bear in mind that the method of general anesthesia will most certainly influence the results and, therefore, need to be mentioned in the material and methods section.

Since we only addressed a small selection of genes in this study, there may still be a set of genes that is not influenced by the paradigms tested. However, we tried to select a set of well-known inflammatory genes and control genes [9]. PPIA and ß2M are highly abundant genes in naïve and ischemic cerebral tissue [18, 19]. Our study was designed to demonstrate the influence of cDNA synthesis on RT efficacy and PCR data and not to test the suitability of single genes to serve as markers to monitor cerebral inflammation.

## Conclusions

We believe that real-time RT-PCR is a magnificent technique for the detection of low-abundant mRNA. The need for a systematic assessment of reference gene validation and normalization strategies has long been recognized and been addressed in numerous publications. Since it has been shown that experimental variation in reverse transcription PCR is mainly attributable to the reverse transcription step [1], a special focus on this critical step is needed. Our study shows that even though (a) the experiment was run in triplicate, starting with the reverse transcription step, (b) an equal total RNA concentration was used in all samples, and (c) the same reverse transcription priming strategy and reaction conditions were applied at all times, the influence of the reverse transcription step clearly reflects on the resulting PCR data. We conclude that expression data obtained from target genes that were normalized with non-regulated control genes cannot be compared with those of other studies if different RT kits are used. To facilitate the comparability of results, we recommend using relative expression ratios by normalizing expression data with naïve sample data.

## Supporting Information

S1 File Data file of the conducted experiments. The file contains the complete raw data set of all experiments in XLSX file format.
(XLSX)
